# Predicting the distribution of suitable habitat of the poisonous weed *Astragalus variabilis* in China under current and future climate conditions

**DOI:** 10.3389/fpls.2022.921310

**Published:** 2022-09-09

**Authors:** Ruijie Huang, Huimin Du, Yuting Wen, Chunyan Zhang, Mengran Zhang, Hao Lu, Chenchen Wu, Baoyu Zhao

**Affiliations:** ^1^College of Veterinary Medicine, Northwest A&F University, Yangling, China; ^2^Institute of Poisonous Plants in Western China, Northwest A&F University, Yangling, China

**Keywords:** locoweed, MaxEnt, climate change, habitat suitability, livestock poisoning control and prevention

## Abstract

*Astragalus variabilis* is a locoweed of northwest China that can seriously impede livestock development. However, it also plays various ecological roles, such as wind protection and sand fixation. Here, we used an optimized MaxEnt model to predict the distribution of suitable habitat of *A. variabilis* under current (1970–2000) conditions and future (2021–2080) climate change scenarios based on recent occurrence records. The most important environmental variables (suitability ranges in parentheses) affecting the distribution of *A. variabilis* were average maximum temperature of February (–2.12–5.34^°^C), followed by total precipitation of June (2.06–37.33 mm), and topsoil organic carbon (0.36–0.69%). The habitat suitability of *A. variabilis* was significantly correlated with the frequency of livestock poisoning (*p* < 0.05). Under current climate conditions, the suitable environment of *A. variabilis* was distributed in central and western Inner Mongolia, Ningxia, central and northwestern Gansu, central and northwestern Qinghai, and the four basins around the Tianshan Mountains in Xinjiang. Under future climate conditions, the suitable habitat of *A. variabilis* shifted to higher latitudes and altitudes. No previous studies have used niche models to predict the suitable environment of this species nor analyzed the relationship between the habitat suitability of poisonous plants and the frequency of animal poisoning. Our findings provide new insights that will aid the prevention of livestock animal poisoning and the control of poisonous plants, promote the development of the livestock husbandry industry, and provide basic information that will facilitate the maintenance of the ecological balance of grassland ecosystems.

## Introduction

According to the 5th Global Climate Change Assessment Report of the United Nations Intergovernmental Panel on Climate Change (IPCC), the world’s average surface temperature has increased by 0.85°C over the past 130 years and is continuing to increase (Stocker, 2013). The effects of warming on the distribution of moisture and the soil microclimate are thought to be particularly important in shaping the range shifts of plants, as their distributions are largely determined by abiotic factors such as precipitation, temperature, soil, and elevation (O’Connor et al., 2020). The effects of climate warming on the distribution of plants have thus been a major focus of research in recent years (Bellard et al., 2012). Studies of how climate change might affect the distribution of plants can aid agricultural production and biodiversity conservation and promote the sustainability of ecosystems.

**FIGURE 1:**
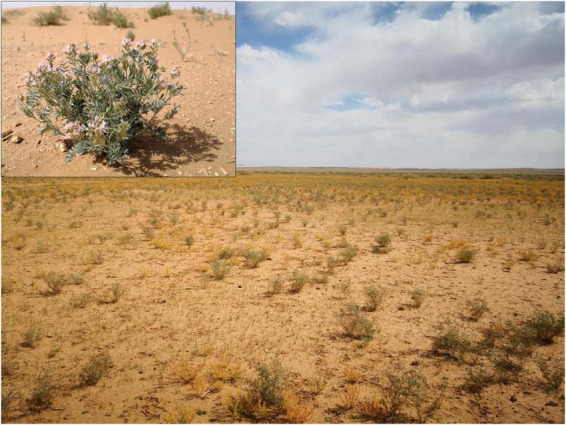
*Astragalus variabilis* (inset) and its habitat in Alxa, Inner Mongolia.

Locoweeds (e.g., *Astragalus*, *Oxytropis*, and *Swainsona*) are poisonous plants that have caused major economic losses to livestock industries in China, Australia, North America, and South America (Lu et al., 2014; Cook et al., 2016; Welch et al., 2018). *Astragalus variabilis* Bunge ex Maxim (Figure 1), a cold-resistant and drought-tolerant locoweed plant, is one of the main threats to the development of the livestock industry in northwest China (Dong et al., 2003). The main toxic component of *A. variabilis* is swainsonine, which can cause neurologic symptoms, reproductive dysfunction, and eventually death in poisoned animals (Wang et al., 2015). Despite its deleterious effects (Zhou et al., 2013), *A. variabilis* is nutritious and can be used as fodder after being detoxified, which could alleviate forage shortages in arid areas (Zhao et al., 2006; Tao et al., 2020). *A. variabilis* can also grow in arid and barren desert steppe, where it can prevent sand fixation, slow soil erosion, and improve soil chemical properties (Zhao et al., 2006; Wang Q. H. et al., 2021). Thus, the development of strategies for managing *A. variabilis* that balance the needs of ecological systems and farmers is needed, and predictions of the potential distribution of *A. variabilis* can provide important information that aids the development of such management strategies.

Species distribution models (SDMs) can be used to predict suitable environments for species by integrating species occurrence data with relevant environmental variables (Guisan and Zimmermann, 2000). Maximum Entropy (MaxEnt) models have become a popular tool for predicting the potential distributions of species because they have been shown to have greater predictive power and accuracy compared with other SDMs (Phillips et al., 2006; Panda and Behera, 2019). MaxEnt models are also capable of handling complex interactions between response and predictor variables, and model outputs remain robust to small sample sizes (Wisz et al., 2008; Elith et al., 2011). Moreover, MaxEnt models are simple to use for researchers in various fields and only require species occurrence data and environmental layers. The information provided by these models has been widely used in the fields of ecology, evolutionary biology, conservation biology, and biosafety (Elith et al., 2011; Luo et al., 2017).

Most previous studies on locoweed plants have focused on examining their deleterious effects, toxic components, poisoning mechanisms, and endophytic fungi (Lu et al., 2012; Yao et al., 2013). However, few studies have examined the ecology of locoweed plants, and no studies to date have attempted to predict the distribution of suitable habitats of locoweeds under different climate scenarios. Here, niche models were used for the first time to predict the distribution of suitable habitat of locoweed plants and analyze the relationship between habitat suitability and the frequency of livestock poisoning. The results of our study will aid future efforts to minimize the deleterious effects of poisonous plants and prevent their spread, promote the development of the livestock husbandry industry in northwest China, and provide basic information that will facilitate the maintenance of the ecological balance of grassland ecosystems.

## Materials and methods

**FIGURE 2:**
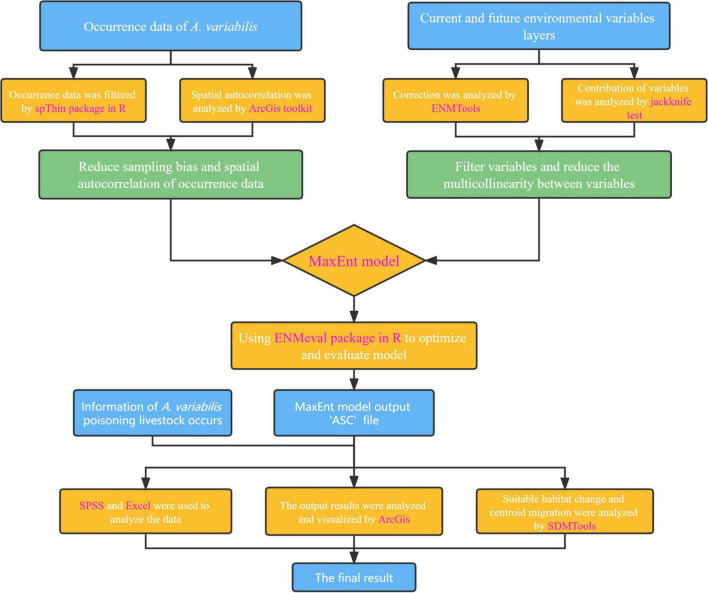
Flow chart of the design of this study. The software, packages, and tools used are indicated in purple font.

The design of this study is summarized in a flow chart in Figure 2. We used the following software, packages, and tools: spThin package (Aiello-Lammens et al., 2015) and ENMeval 2.0 package (Kass et al., 2021) in R 4.2.1, ENMTools (Warren et al., 2010), MaxEnt 3.4.4 (including the jackknife test function) (Phillips et al., 2006), SDMTools 2.0 (Brown et al., 2017), ArcGIS 10.7 (including its various toolkits), and SPSS 20.0.

### Target species and study area

*Astragalus* L. (Fabales: Fabaceae: Galegeae) is mainly distributed in arid and semi-arid regions of the northern hemisphere and temperate South America (Podlech, 1986; Ranjbar and Karamian, 2003). China is one of the major centers of *Astragalus* diversity and has more than 400 species belonging to 59 sections (Huang et al., 2018). *A. variabilis* belongs to the subgenus *Cercidothrix* Bunge, A. Sect. *Craccina* (Stev.) Bunge, and it is mainly distributed in Inner Mongolia, Ningxia, Gansu, and Qinghai (Editorial Committee of Chinese Flora, C.A.O.S., 2006). *A. variabilis* is known to be resistant to cold, drought, and barren soils (Dong et al., 2003).

We conducted a preliminary study in which we determined the area of the potential distribution of *A. variabilis* in a restricted area: 30.04°–49.35°N, 73.45°–118.86°E. The study area covers most pastoral areas in China, including central and western Inner Mongolia, Ningxia, Gansu, Qinghai, Xinjiang, central and northern Tibet, and northern Sichuan. Most of this preliminary study region comprises arid and semi-arid regions that receive low amounts of precipitation in an uneven fashion (annual precipitation ranges from 200 to 400 mm) and experience rapid changes in temperature and precipitation (Lv et al., 2009).

### Species occurrence data

We obtained records of *A. variabilis* occurrence from fieldwork conducted between 2014 and 2021; the coordinates of all localities were taken using a GPS (GARMIN GPSMAP 621sc; accuracy ± 3 m). Records were also obtained from searches conducted on two online databases: the Chinese Virtual Herbarium^1^ and the Global Biodiversity Information Facility.^2^ To ensure the accuracy of occurrence points, we only used records with precise coordinates after 2000. All records were carefully inspected, and records with erroneous data and duplicate records were removed. See Supplementary Table 1 for details of all records. One assumption of MaxEnt models is that all areas have been systematically or randomly sampled (Phillips et al., 2009). However, samples are often spatially autocorrelated due to sampling bias, and this can result in the overfitting of MaxEnt models and affect the model’s predictive ability (Warren et al., 2014; Kong et al., 2019). Spatial filtering of occurrence points has been shown to be effective for reducing spatial autocorrelation and sampling bias (Kong et al., 2019). Thus, we used the spThin package in R to spatially filter the occurrence points (Aiello-Lammens et al., 2015) at distances of 5, 10, and 20 km. We used AcrGIS to conduct spatial autocorrelation analysis on the unfiltered data as well as the data that were spatially filtered at 5, 10, and 20 km (Jiang et al., 2022). These data were added to the MaxEnt model, and the degree of overfitting was evaluated using a 10% training omission rate (OR10) (Radosavljevic and Anderson, 2014). The distance of OR10 to 0.1 is positively correlated with the overfitting degree of the model (Muscarella et al., 2014; Radosavljevic and Anderson, 2014). Finally, the optimal spatial filtering distance was selected.

### Environmental variables

Because few ecological studies of *A. variabilis* have been conducted, we used the environmental variables most commonly used in previous studies that have predicted the distribution of suitable habitat for various plant species (Fois et al., 2018; Shi et al., 2022; Zhang L. et al., 2022). Given that we were interested in comparing current and future suitable habitats, environmental variables with both current and future data available were used. We initially used 19 bioclimatic, 36 climatic variables, 1 topographical variable, and 4 soil variables that might affect the distribution of *A. variabilis* (Supplementary Table 2). Bioclimatic, climatic, and topographical layers were downloaded from WorldClim 2.1^3^ (Fick and Hijmans, 2017), and soil variable layers were downloaded from the Harmonized World Soil Database version 1.2 (HWSD v1.2)^4^ (Wieder et al., 2014). The spatial resolutions of the environmental variable layers from WorldClim were 10 arc min, 5 arc min, 2.5 arc min, and 30 arc s. We used the 2.5 arc-min spatial resolution layers for analyses. There were no observable differences in the results when data at a resolution of 30 arc s and 2.5 arc min were used, which is consistent with the results of a previous study (Guisan et al., 2007). The processing of the data is also much more rapid when 2.5 arc-min data are used compared with 30 arc-s data. Data at a resolution of 2.5 arc min have been used in several previous studies that have estimated the potential distributions of species (Wan et al., 2021; Xu et al., 2021; Jiang et al., 2022).

Future climate data were derived from four shared socioeconomic pathways (SSP126, SSP245, SSP370, and SSP585) under the BCC-CSM2-MR model from the Coupled Model Inter-comparison Project 6 (CMIP6) published by the IPCC on the WorldClim website (Fick and Hijmans, 2017; Riahi et al., 2017). Previous studies have shown that the BCC-CSM2-MR model is robust for modeling climate change in China (Yang et al., 2016; Shi et al., 2020). SSP126, SSP245, SSP370, and SSP585 represent future climate scenarios with low to high carbon emissions (Table 1; Riahi et al., 2017). Future climate prediction was carried out in three periods: 2021–2040 (2030s), 2041–2060 (2050s), and 2061–2080 (2070s). We included the same elevation and soil layers in both the current and future climate models given that elevation and soil can be expected to remain unchanged over these short time frames.

**TABLE 1:** Description of four shared socioeconomic pathways.

Future climate scenarios	Social development description	CO_2_ emissions and climate change description
SSP126	Societies shift to more sustainable practices, with a shift in focus from economic growth to overall well-being, investment in education, and health; inequality decreases.	Global CO_2_ emissions are cut drastically, reaching net zero after 2050. By the end of the century, the global temperature increase stabilizes to ca. 1.8°C.
SSP245	This is a “middle-of-the-road” scenario, in which socioeconomic factors follow their historical trends without significant change. Progress in sustainability is slow, and development and income growth are uneven.	CO_2_ emissions hover around current levels before starting to decline by mid-century but do not reach net zero by 2100. By the end of the century, the global temperature increases by 2.7°C.
SSP370	Competition among nations intensifies, and a global focus on national security and food security predominates.	Emissions and temperatures rise steadily; by 2100, CO_2_ emissions roughly double from current levels. By the end of the century, the average global temperature increases by 3.6°C.
SSP585	This is a future to be avoided at all costs. The global economy grows rapidly, but this growth is driven by the extraction of fossil fuels and an energy-intensive lifestyle.	By 2050, CO_2_ emissions roughly double. By the end of the century, the global average temperature increases by 4.4°C or higher.

Because multicollinearity among environmental variables can affect model outputs and result in an over-fitted model, Pearson’s correlation analysis was used to examine correlations among the environmental variables in ENMTools (Sillero, 2011; Li et al., 2022). If the absolute value of the correlation coefficient between two environmental variables was greater than 0.7, the one with a lower percent contribution (according to a jackknife test) was removed (Zeng et al., 2016; Farrell et al., 2019).

### Model optimization and evaluation

Feature class (FC) combinations and regularization multiplier (RM) settings are important parameters affecting the predictions of MaxEnt models (Phillips and Dudik, 2008), and default settings are typically not optimal (Muscarella et al., 2014). We thus used the ENMeval package in R to optimize and evaluate models, which supports more evaluation metrics in its version 2.0 (Kass et al., 2021). Eight RMs (0.5–4 with an interval of 0.5) and six different FC combinations (L, LQ, H, LQH, LQHP, LQHPT, where L = linear, Q = quadratic, H = hinge, P = product, and T = threshold) were used to create 48 (8 RM × 6 FC) candidate models. We used random k-fold cross-validation to verify models (Boria and Blois, 2018; Kass et al., 2021). A recent study suggests that the value of k in k-fold cross-validation should be the natural logarithm of the sample number (Jung, 2018). Here, our sample number was approximately 200; thus, *k* = 5. The optimal model had a low OR10 relative to the default, and the lowest Akaike information criterion (AICc) (Liu et al., 2017; Galante et al., 2018).

Metrics such as the area under the receiver operating curve (AUC), continuous Boyce index (CBI), OR10, and true skill statistic (TSS) were used to evaluate the performance of the model. AUC is a threshold-independent metric that is often used to evaluate the performance of MaxEnt models. The performance of the model was evaluated using the following criteria: fair (0.7 < AUC < 0.8), good (0.8 < AUC < 0.9), and excellent (0.9 < AUC < 1) (Jimenez-Valverde, 2012; Shi et al., 2021). The CBI is also often used to evaluate the transferability and performance of presence–absence models; it is a reliable measure of presence-only-based predictions (Hirzel et al., 2006). CBI ranges from –1 to 1, and values closer to 1 indicate models with higher consistency with the distribution of presence data (Boyce et al., 2002). TSS ranges from –1 to 1, and TSS values closer to 1 indicate higher prediction accuracy of the model (Allouche et al., 2006). When TSS is greater than 0.75, model performance is very good (Landis and Koch, 1977; Franco et al., 2022).

MaxEnt 3.4.4 (Phillips et al., 2006) was used to model the potential distribution of *A. variabilis*. We used optimized FC and RM parameters. The settings selected were as follows: “Create response curves,” “Do jackknife to measure variable importance,” “Random seed,” “Write plot data,” “Write background predictions,” “randomly sample 10,000 background points as pseudo-absences,” “Replicated run type crossvalidate,” “Replicates 5,” and “Output format logistic.” The rest of the settings were default. The MaxEnt model eventually outputs an “ASC” file in logistic output format for each grid.

### Habitat suitability classification

We used ArcGIS to reclassify and visualize the ASC files. The maximum training sensitivity plus specificity threshold (MTSPS) was used to classify the model output results (logistic output) into unsuitable and suitable for *A. variabilis*, which is considered simple and effective (Aidoo et al., 2022). MTSPS has been shown to be robust for selecting thresholds when model outputs are based on presence-only data (Liu et al., 2013). It is less affected by the occurrence:background point ratio and species prevalence, which reduces omission errors for low-prevalence species and misclassification errors for high-prevalence species (Liu et al., 2016). The habitat suitability of *A. variabilis* was reclassified into four categories according to the output logistic value: unsuitable (0–MTSPS), low suitability (MTSPS–0.4), moderate suitability (0.4–0.6), and high suitability (0.6–1.0) (Zhang et al., 2018; Mahatara et al., 2021).

### Analysis of livestock poisoning

We compiled a data set of *A. variabilis* poisoning livestock incidents by conducting a (1) literature review, (2) searching various news outlets, and (3) conducting field studies from 2000 to 2022 (Supplementary Table 3). Livestock poisoning has occurred in the study area for many years, and we recorded the frequency of poisoning incidents by tallying the number of years in which poisoning incidents have been reported. For example, records of animal poisoning were documented in Alxa Left Banner of Inner Mongolia in 2003, 2004, and 2005; thus, a value of 3 was recorded in our data set for Alxa Left Banner. For localities with accurate location descriptions, we used the Baidu coordinate system^5^ to obtain latitude and longitude data, and the logistic output of the location was extracted using the coordinates. For records with imprecise locality descriptions (county level and above), we used the average logistic values over the entire area over which the poisoning incident occurred. Habitat suitability for each poisoning incident was inferred according to logistic output values. Finally, SPSS 20.0 was used to analyze the correlation between habitat suitability and the frequency of poisoning incidents.

### Analysis of changes in the area of suitable habitat

We used ArcGIS and Microsoft Excel to conduct spatial data analyses. The SDM toolbox was used to compare differences in the area of suitable habitat of *A. variabilis* under current and future climate conditions (Brown et al., 2017). The model output results were divided into binary maps using the MTSPS threshold (logistic output values greater than MTSPS were assigned a value of 1, and values less than 1 were assigned a value of 0). Changes were quantified by subtracting the current binary map from the binary maps of each of the four future periods. Grid values ranging from 0 to 1 indicate expansions in the range of suitable habitat; values ranging from 1 to 0 indicate contractions in the range of suitable habitat; and a value of 1 indicates no change in the area of suitable habitat. A value of 0 indicates that the habitat is not suitable (i.e., no occupancy). Similarly, the centroid of the area of suitable habitat in each period was calculated using the SDM toolbox, and the changes in the area of suitable habitat can be visualized by comparing the centroids among periods (Cong et al., 2020).

## Results

### Species occurrence records and environmental variables

A total of 273 occurrence records of *A. variabilis* were obtained through our field investigation, and 9 occurrence records that met our quality requirements were obtained from databases (CVH: 9; GBIF: 0). The results of the spatial autocorrelation analysis and OR10 values of the unfiltered data and data spatially filtered at three different distances are shown in Table 2. Unfiltered data were spatially autocorrelated, as the distribution of these data significantly differed from a random distribution (*p* < 0.01, z > 2.58). There were no significant differences in the distributions of the data spatially filtered at three different distances and random distributions (*p* > 0.10, –1.65 < z < 1.65). Spatial filtering reduces spatial autocorrelation and sampling bias. We used the data that were spatially filtered at a distance of 10 km because the OR10 was lowest for these data. A total of 189 occurrence records were used for modeling.

**TABLE 2:** Spatial autocorrelation and OR10 of occurrence records that were spatially filtered at different distances.

Filter distance	Moran’s *I*	*p*-value	z-score	OR10
Unfiltered	0.1310	0.0000	47.5329	0.1596 ± 0.0552
5 km	–0.0017	0.5264	–0.6351	0.1542 ± 0.0406
10 km	–0.0014	0.6175	–0.4994	0.1481 ± 0.0756
20 km	–0.0009	0.7418	–0.3295	0.1911 ± 0.0457

After reducing the multicollinearity among environmental variables, a total of 10 environmental variables (elev, bio7, bio15, bio19, prec6, tmax2, t_oc, t_teb, t_ph, and t_tex) were retained for modeling. Correlations among the environmental variables used in the final model were all less than 0.7 (Supplementary Figure 1).

### Model optimization and evaluation

The MaxEnt model uses all feature types by default when there are more than 80 training samples, and the regularization constant is 1 (FC = LQHPT, RM = 1) (Phillips et al., 2006; Phillips and Dudik, 2008). Optimization using the ENMeval package revealed that the AICc value of the model was lowest (delta AICc = 0) when FC = H and RM = 0.5. The avg.AUC, avg.CBI, and avg.TSS were higher and the avg.OR10 was lower for the optimized model compared with the default model (Table 3). These findings indicate that FC = H and RM = 0.5 reduced the overfitting and complexity of the MaxEnt model and enhanced the transferability of the model. In addition, AUC > 0.9, TSS > 0.75, and CBI was close to 1 for the optimized model. This indicates that the performance and transferability of the model were high. We also added the occurrence points removed by spatially filtering that were not used for modeling into the current suitable habitat simulation map to evaluate their overlap with the predicted suitable area (Supplementary Figure 2). A total of 92 of the 93 occurrence records (98.92%) were located in suitable habitat, and one occurrence record was located in unsuitable habitat but was close to an area with suitable habitat. Thus, this model was sufficiently robust for modeling the potential distribution of *A. variabilis*.

**TABLE 3:** Comparison of metrics before and after model optimization.

Model	Model parameter	Avg. AUC	Avg. CBI	Avg. OR10	Delta.AICc	Avg. TSS
Default	FC = LQPH, RM = 1	0.9551 ± 0.0099	0.8734 ± 0.0508	0.1539 ± 0.0533	28.9002	0.8219 ± 0.0085
Optimized	FC = H, RM = 0.5	0.9582 ± 0.0075	0.9016 ± 0.0446	0.1427 ± 0.0508	0.0000	0.8227 ± 0.0127

### Key environmental variables affecting the distribution of *Astragalus variabilis*

**FIGURE 3:**
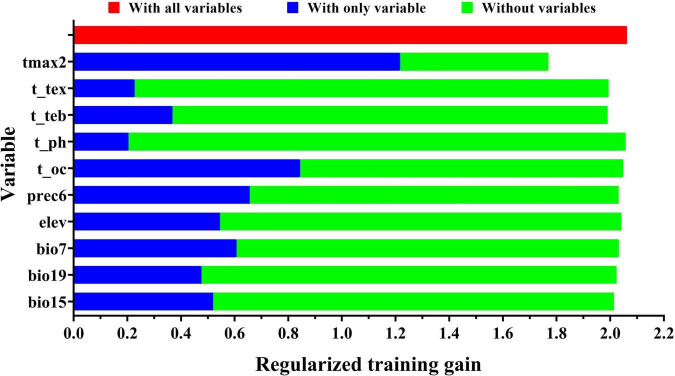
The relative importance of environmental variables in determining the potential distribution of *A. variabilis* according to jackknife tests.

The contribution rates of 10 environmental variables are shown in Table 4. The variables with the highest contribution rates to the model were tmax2, bio7, and prec6, which had a cumulative contribution rate of 64.1%. The results of jackknife tests of variable importance are shown in Figure 3. The environmental variable with the highest training gain when used in isolation was tmax2, followed by t_oc and prec6; thus, tmax2, t_oc, and prec6 were the most informative variables. The environmental variable that decreased the gain the most when it was omitted was also tmax2; tmax2 thus provided the most information that was not provided by the other variables. In sum, the key environmental factors affecting the distribution of *A. variabilis* were tmax2, prec6, and t_oc.

**TABLE 4:** Details of environmental variables used for modeling.

Environmental variables	Description	Percent contribution	Suitable ranges	Most suitable environmental values	Highest habitat suitability (logistic value)
bio7	Annual temperature range	13.9%	37.47–66.46^°^C	45.76^°^C	0.62
bio15	Precipitation seasonality	3.1%	30.58–111.49	100.04	0.67
bio19	Precipitation of coldest quarter	10.1%	0–12.54 mm	4.08 mm	0.56
prec6	June total precipitation	10.6%	2.06–37.33 mm	11.88 mm	0.66
tmax2	February average maximum temperature	39.6%	–2.12–5.34^°^C	1.69^°^C	0.66
elev	Elevation	4.2%	542.64–3525.91 m	1362.65 m	0.66
t_oc	Topsoil organic carbon	0.7%	0.36–0.69% weight	0.50% weight	0.65
t_teb	Topsoil total exchangeable bases	9.4	0–50.67 cmol/kg	38.03 cmol/kg	0.93
t_ph	Topsoil pH (H_2_O)	0.4	0–9.79 –log(H +)	7.85 –log(H +)	0.76
t_tex	Topsoil USDA texture	8.1	2, 3, 4, 5, 7, 9, 10, 11, 12, 13	12	0.78

**FIGURE 4:**
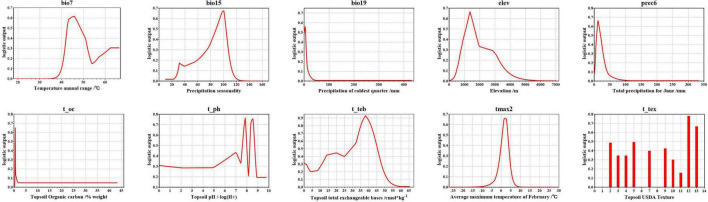
Logistic output of the final model of *A. variabilis* vs. the environmental variables used in the model.

The environmental variable response curves indicate the degree to which each environmental variable affects the model output result (logistic output) and thus the habitat suitability of *A. variabilis* (Figure 4). The logistic value ranges from 0 to 1, and values closer to 1 indicate higher habitat suitability. Values of the environmental variables are considered suitable for *A. variabilis* when the logistic output exceeds the MTSPS. The suitable ranges, most suitable values of environmental variables, and highest habitat suitability values are shown in Table 4. With the exception of t_ph and t_tex, the response curves of all variables were unimodal or nearly unimodal; however, the response curve for t_ph was approximately bimodal (two higher peaks at ph = 7.85 and 8.54). The suitable topsoil USDA textures (t_tex) included silty clay (code 2), clay (code 3), silty clay loam (code 4), clay loam (code 5), silt loam (code 7), loam (code 9), sandy clay loam (code 10), sandy loam (code 11), loamy sand (code 12), and sand (code 13). Habitat suitability was highest when the soil type was loamy sand, followed by sand, silty clay, and clay loam.

### Current suitable habitat and livestock poisoning incidents of *Astragalus variabilis*

**FIGURE 5:**
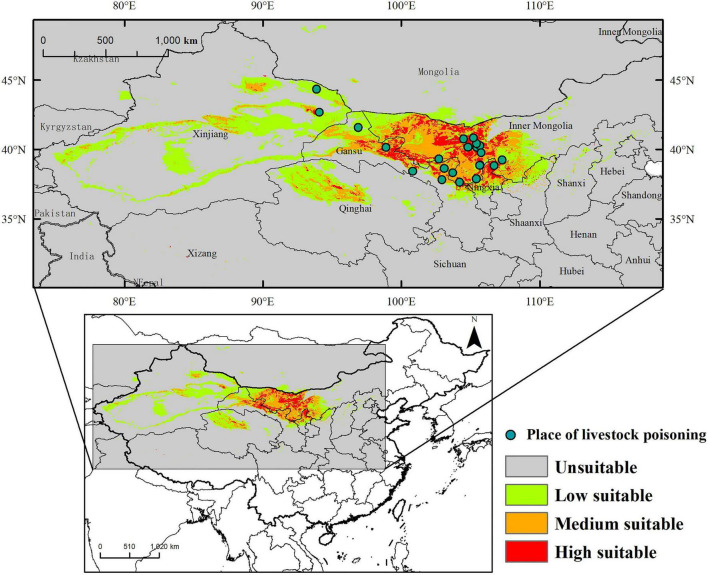
Distribution of the suitable habitat of *A. variabilis* in China under current climate conditions and the locations of livestock poisoning incidents.

The suitable habitat of *A. variabilis* under current climate conditions is shown in Figure 5. The area of suitable habitat was approximately 1.04 × 10^6^ km^2^, which is 12.72% of the study area. The area of low, moderate, and high suitability habitats was 6.31 × 10^5^, 2.88 × 10^5^, and 1.26 × 10^5^ km^2^, which comprises approximately 7.70, 3.49, and 1.52% of the study area, respectively. Suitable habitats were mainly observed in Central and Western Inner Mongolia, including Alxa League, Bayannaoer, and Ordos; the center and edge of the Tarim Basin, around the Tianshan Mountains, the southeastern part of Junggar Basin, Turpan Basin, Hami Basin, and the northern part of Barkun Grassland in Xinjiang; Central and Northwest Gansu including Jiuquan City, Zhangye City, Jinchang City, Wuwei City, Baiyin City, and Zhongwei City; Central and Northwest Qinghai (Haixi Mongolian Autonomous Prefecture); and Ningxia Province. Suitable habitats were also observed in Shaanxi, Shanxi, Hebei, Sichuan, and Xizang Provinces.

We determined the frequency of livestock poisoning incidents caused by *A. variabilis* in areas varying in habitat suitability across the study period (Table 5 and Supplementary Table 4). The locations of livestock poisoning incidents are marked in Figure 5. Pearson correlation analysis showed that the frequency of poisoning was significantly positively correlated with habitat suitability (logistic output) (Pearson correlation coefficient = 0.96, *p* < 0.05).

**TABLE 5:** Frequency of livestock poisoning incidents caused by *A. variabilis* by the degree of habitat suitability.

Habitat suitability	Logistic output interval	Frequency
Unsuitable	0–0.1388	0
Low suitable	0.1388–0.4	12
Medium suitable	0.4–0.6	30
High suitable	0.6–1.0	35

### Suitable habitat of *Astragalus variabilis* under future climate conditions

**FIGURE 6:**
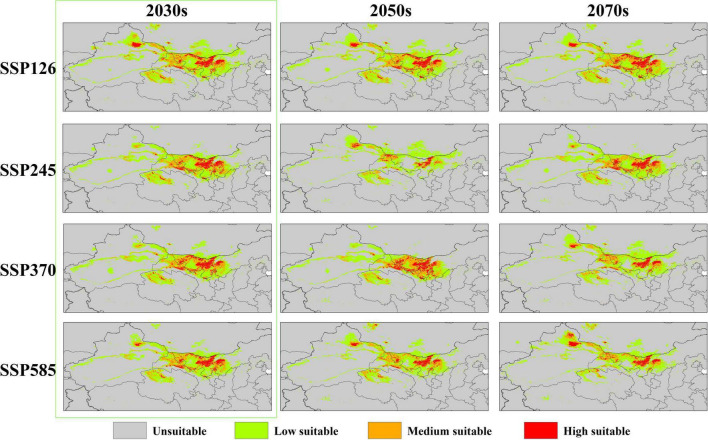
The suitable habitat of *A. variabilis* in China under current climate conditions.

**FIGURE 7:**
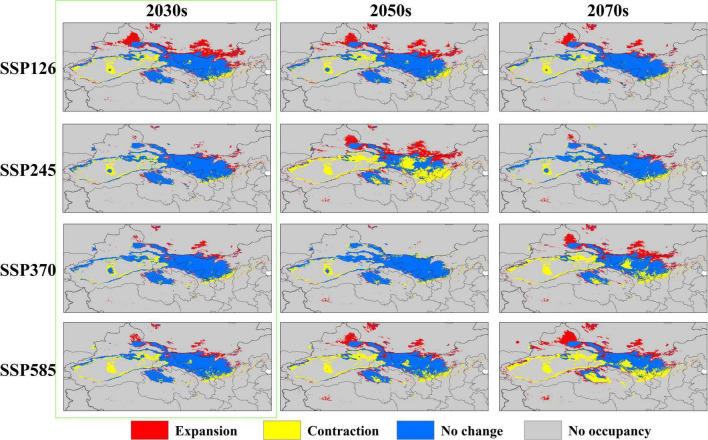
Changes in the area of suitable habitat of *A. variabilis* in China under different future climate conditions relative to that under current climate conditions.

**FIGURE 8:**
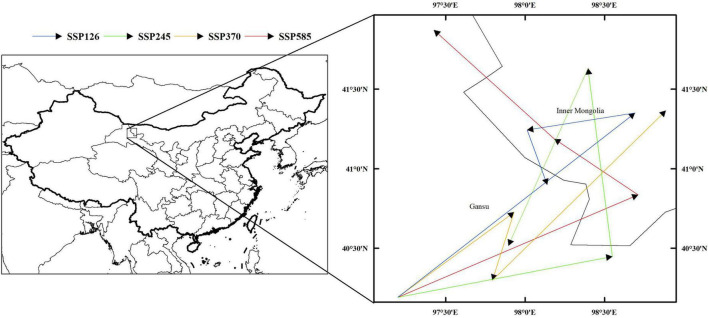
Migration of the centroid of suitable habitat of *A. variabilis* in China.

**FIGURE 9:**
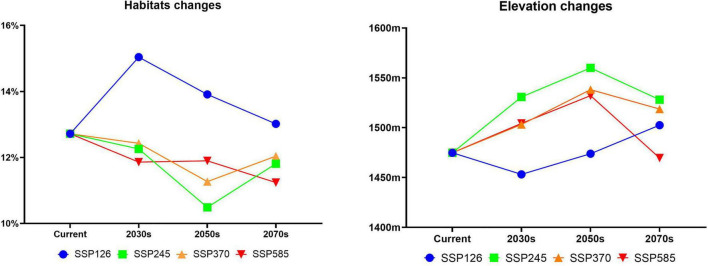
Changes in the relative proportions and average elevation of suitable habitat of *A. variabilis* in China.

The distribution of the suitable habitat of *A. variabilis* under different future climate scenarios is shown in Figure 6. To compare the distribution of suitable habitat in the future with the current distribution of suitable habitat, we analyzed changes in the geographical distribution of suitable habitats, including the expansion and contraction of suitable habitats (Figure 7) and the movement of the centroid of suitable habitats over the study period (Figure 8). The area of suitable habitat was predicted to be reduced to varying degrees in the south, including the Tarim Basin, Turpan Basin, and Hami Basin in Xinjiang; Wuwei City and Baiyin City in Gansu; and southern Haixi Mongolian Autonomous Prefecture in Qinghai; central Ningxia; and northern Shaanxi. The area of suitable habitat was predicted to expand to varying degrees in the north, including the Ili River Valley and the Junggar Basin in Xinjiang; northern Bayannaoer, northern Baotou, and northern Ulanqab in Inner Mongolia; and Harwusu Lake and South Gobi Province in Mongolia. Under SSP126 and SSP245, the centroid of suitable habitat first migrated to the northeast and then returned to the southwest; under SSP370, the centroid migrated to the northeast; and under SSP585, the centroid first migrated northeast and then to the northwest. We also characterized changes in the proportion of habitats varying in suitability in the study area and the average elevation in areas with suitable habitat (Figure 9). Under SSP126, the area of suitable habitat first increased and then decreased; under SSP245, SSP370, and SSP585, the area of suitable habitat decreased. The average elevation of suitable habitat generally increased under the different climate scenarios. The relative proportions of habitats varying in suitability are shown in Supplementary Figure 3.

## Discussion

*Astragalus variabilis* is one of the main locoweeds that induces significant losses to the animal husbandry industry in northwest China. In this study, we used optimized MaxEnt models to (1) predict the distribution of suitable habitat of *A. variabilis* under current and future climate conditions; (2) analyze the key environmental variables affecting the distribution of *A. variabilis*; and (3) analyze the correlation between the occurrence of animal poisoning and habitat suitability for *A. variabilis*. The findings of this study provide valuable information that will aid the management of *A. variabilis*.

### Interpretation of experimental results

In studies aimed at predicting the area of suitable habitat of species, only models with good performance have high confidence. The main methods currently used for evaluating the robustness of MaxEnt models include threshold-independent measurement, threshold-dependent measurement, model transfer performance, and visual evaluation (Kong et al., 2019). The use of multiple evaluation metrics as opposed to a single evaluation metric is becoming increasingly common for evaluating the performance of SDMs (Lei et al., 2015). We used AUC, TSS, CBI, visual evaluation, and other metrics to comprehensively evaluate the robustness of our model, and all of these metrics indicated that our model was reliable.

Environmental factors affect plant growth and development and also restrict the distribution of plant species (Thuiller, 2007). Previous studies have shown that *A. variabilis* is highly adaptable to arid environments with low temperatures and barren soils (Dong et al., 2003; Yao et al., 2013; Wang Q. H. et al., 2021). In this study, the response curves indicated that *A. variabilis* is well adapted to environments with low precipitation (bio19, prec6), cold (tmax2), and nutrient-poor soils (t_oc, t_teb). According to the *Flora of China*, *A. variabilis* grows on dry riverbeds or Gobi sandy soils in desert areas ranging from 900 to 3,100 m in elevation (Editorial Committee of Chinese Flora, C.A.O.S., 2006). This is generally consistent with our suitable ranges for altitude and soil type. The overall response curve of t_ph was above the MTSPS threshold, which might stem from the fact that the contribution rate of t_ph to the model was low; consequently, the response curve may not consistent with actual observations. However, this also indicated that *A. variabilis* is tolerant of soil acidity and alkalinity. In addition, the response curves of bio7 and bio15 revealed that *A. variabilis* is highly tolerant of high annual mean temperature differences and precipitation seasonality. No studies to date have evaluated the effects of soil pH, annual mean temperature difference, and precipitation seasonality on *A. variabilis*; thus, additional studies are needed to confirm these possibilities.

Suitable habitat of *A. variabilis* under current climate conditions was mainly distributed in central and western Inner Mongolia, Ningxia, central and northwestern Gansu, central and northwestern Qinghai, and the four basins around the Tianshan Mountains in Xinjiang. These findings are consistent with the known geographical distribution of *A. variabilis* (Zhou et al., 2013; Guo et al., 2021). Global climate change (including changes in temperature and precipitation patterns) has induced major changes in the growth, morphological characteristics, distribution, and area of global vegetation (Rosenzweig et al., 2008). Several studies have examined the impact of climate change on species distributions. More than half of Europe’s 1,350 plant species are expected to be vulnerable or threatened by the 2080s due to climate change (Thuiller et al., 2005). In recent decades, a northward expansion of various plant species has been observed in Europe, including an increase in the abundance of thermophilic plant species compared with 30 years ago and a marked decrease in hardy plant species, which most likely stems from warmer temperatures (Woldearegay, 2020). In Vermont, United States, the limits of the northern hardwood–boreal forest ecotone moved 91–115 m upslope between 1962 and 2005 (Beckage et al., 2008). In Canada, the suitable habitats for four tree species are predicted to shift significantly to higher latitudes and altitudes (Flower et al., 2013). *Leymus racemosus* is distributed in the Junggar Basin, Xinjiang, China. The area of suitable habitat of this species is expected to decrease in the future, and its distribution is expected to move northwest and to higher altitudes (Zhang R. et al., 2022). A decrease in suitable habitat in China over the next 80 years and a northward shift in the area of suitable habitat have also been predicted for *Astragali radix*, a plant distributed in arid and semi-arid areas of China and with similar growth characteristics to *A. variabilis* (Peng and Guo, 2017). Our results show that the suitable habitat of *A. variabilis* will move to high latitudes and high altitudes under various future climate scenarios. This is consistent with previous research suggesting that the distributions of most species will shift to higher latitudes and elevations as global warming intensifies (Lenoir et al., 2008). Temperature is the most important environmental factor affecting the distribution of *A. variabilis* (Figure 3 and Table 4). Under future global warming, the distribution of *A. variabilis* is predicted to migrate to high latitudes and high altitudes because the temperatures in these regions are lower compared with regions at low latitude and low alitutude. The area of suitable habitat of *A. variabilis* is predicted to decrease under all scenarios with the exception of SSP126. This might stem from variation in the magnitude of climate change under different SSP scenarios. Carbon emissions are lowest under SSP126 among all scenarios, and the magnitude of change in precipitation patterns was relatively insignificant. The increase in temperature and precipitation in northwest China due to climate change (Wang C. H. et al., 2021) likely explains the decrease in suitable habitat, as *A. variabilis* prefers dry environments and cooler temperatures. The reduction in the area of suitable habitat for *A. variabilis* could be a benefit to the livestock industry, as this would result in fewer livestock poisoning incidents. However, this would only provide a benefit if other suitable forages could replace *A. variabilis* in areas where it is expected to disappear. Indeed, an absence of ecologically equivalent species to replace *A. variabilis* in such areas might exacerbate the desertification of desert steppe.

### Practical implications

Locoweed is one of the main poisonous weeds impeding the development of the animal husbandry industry, especially in developing countries, as livestock poisoning incidents have been frequently reported in recent years (Cook et al., 2017; Martinez et al., 2019; Reis et al., 2019). This might stem at least in part from the lack of investment by local governments in the control of poisonous weeds. The US livestock industry lost hundreds of millions of dollars to livestock poisoning caused by poisonous weeds in the 1980s (Panter et al., 2007). Subsequently, the United States government invested heavily in campaigns aimed at the control of poisonous weeds and established the Institute of Poisonous Plants, which specializes in the study of poisonous weed species in natural grasslands, assessment of toxic disasters, and research on technology for the diagnosis and control of poisoning diseases (Welch et al., 2012; Fu et al., 2019). As a consequence of these efforts, there are now few reports of livestock poisoning incidents in the United States. For countries with less capital for investment, controlling high-risk areas could be a cost-effective approach. Historical records and field investigations are the only tools available in China for the identification of priority areas for focused control efforts. MaxEnt models have been widely used in recent years for rare species requiring protection (Cao et al., 2020), the control of pests and diseases (Wakie et al., 2020), and the control of invasive species (Herrando-Moraira et al., 2020). These models, which can be developed using freely available software, require only occurrence records and environmental data to make predictions. This software is relatively user-friendly. Recently, the habitat suitability output of the MaxEnt model was confirmed to be related to the alkaloid content in medicinal plants, and this information can be used to enhance the quality of medicinal plants (Li et al., 2020). We hoped to use the MaxEnt model to prevent livestock poisoning incidents caused by *A. variabilis*. This hypothesis predicts a correlation between animal poisoning and habitat suitability. The results of the correlation analysis supported this hypothesis, as the frequency of livestock poisoning incidents was higher in areas with higher habitat suitability.

Many studies have investigated approaches to prevent the poisoning of livestock by *A. variabilis*. For example, physical and chemical approaches can be used to eliminate *A. variabilis*; however, these control methods can have deleterious effects on the environment (Yao et al., 2013). Biological methods that involve facilitating the colonization of other plants to replace *A. variabilis* have also been explored; however, this approach is difficult to implement, and it can often take a long time before positive effects are observed (Ralphs et al., 2007). The administration of preventive medicine for livestock in high-risk areas is a simple and effective strategy (Zhao et al., 2010). We used the next 20 years (2030s) as an example for discussing the prevention and control of livestock poisoning. Changes in the area of suitable habitat of *A. variabilis* were observed in the 2030s under all four SSP scenarios, as indicated by the green marked boxes in Figures 6, 7. The least favorable scenario was SSP124; under this scenario, suitable habitats expanded greatly in the Jungar Basin in Xinjiang and Bayannaoer, Baotou, and Ulanqab in Inner Mongolia. Highly suitable habitats were widely distributed in the Jungar Basin of Xinjiang and Alxa Left Banner and Bayannaoer City in Inner Mongolia. More preventive measures will need to be implemented in the pastoral areas of the Jungar Basin in Xinjiang and Bayannaoer City in Inner Mongolia in the 2030s. The prevention and control of livestock poisoning has always been a major focus in the Alxa Zuoqi area of Inner Mongolia. We propose that a locoweed detoxification site be established in the Alxa Zuoqi area because this area has long had abundant *Astragalus* resources, and pasture is often lacking due to drought. *A. variabilis* can be harvested for detoxification and used as feed, whereas other drought-tolerant plants can be artificially planted to improve the grassland environment.

### Limitations and prospects

MaxEnt models can predict the suitable distribution of species under future climate change scenarios; it is thus an important tool for simulating the distribution of suitable habitats of species (Zhao et al., 2021). Although MaxEnt models have high accuracy, this does not mean that the predicted suitable area is always completely consistent with the actual distribution of species (Gebrewahid et al., 2020). The distribution of poisonous weeds is not only affected by climate, topography, and soil factors but also by other factors such as reproduction and pollination type, interactions between species, social development, and human activities. With our current technology, some of these factors are difficult to quantify; others cannot be forecasted and thus incorporated into models. In addition, predictions of future events are always vulnerable to some degree of uncertainty, and the error in predictions is related to how far in the future predictions are made. Changes in suitable habitat merit increased attention in habitat modeling studies. Despite their many assumptions and uncertainties, SDMs are a key tool for predicting the area of suitable habitat of species under future conditions (Wiens et al., 2009).

In future studies, a greater number of environmental factors lacking future data, such as the normalized vegetation index (NDVI), solar radiation, wind speed, and water vapor pressure, can be used to build models to predict the current distribution of suitable habitat and evaluate the consistency between the predicted and actual distribution of species. How the distributions of other locoweed species in natural grasslands might be altered under future climate change remains unclear. The similarity in the ecological niches of these locoweed plants also requires clarification.

## Conclusion

Our optimized MaxEnt model made robust predictions of the suitable habitat of *A. variabilis*. The average maximum temperature of February (tmax 2), precipitation of June (prec 6), and topsoil organic carbon (t_oc) were the most important environmental variables affecting the area of suitable habitat, which is consistent with its cold resistance, drought tolerance, and tolerance for barren soils. The area of suitable habitat of *A. variabilis* was predicted to decrease under all scenarios with the exception of SSP126. Under the four SSP climate scenarios, the suitable habitat of *A. variabilis* is predicted to shift to higher latitudes and altitudes. Our findings have implications for the prevention and control of poisonous plants as well as the maintenance of the ecological balance of grassland ecosystems.

## Data availability statement

The original contributions presented in this study are included in the article/Supplementary material, further inquiries can be directed to the corresponding author/s.

## Author contributions

BZ contributed to the study conception and design, agreement to be accountable for all aspects of the work related to the accuracy and integrity of any part of the work, and approval of the final version. RH substantially contributed to the study conception and design, data acquisition, analysis, and interpretation, and drafting and revision of the manuscript for intellectual content. HD, YW, CZ, and MZ contributed to data acquisition and analysis. HL contributed to revising the manuscript. CW contributed to agreement to be accountable for all aspects of the work related to the accuracy or integrity of any part of the work. All authors contributed to the article and approved the submitted version.

## Footnotes

1
https://www.cvh.ac.cn/

2
https://www.gbif.org/

3
https://www.worldclim.org/

4
https://daac.ornl.gov/

5
http://api.map.baidu.com/lbsapi/getpoint/
