# Supplementary figures and images for: Predicting the distribution of suitable habitat of the poisonous weed Astragalus variabilis in China under current and future climate conditions
Source: Front Plant Sci. 2022 Sep 9;13:921310. doi: 10.3389/fpls.2022.921310 (PMC9531759; doi:10.3389/fpls.2022.921310)

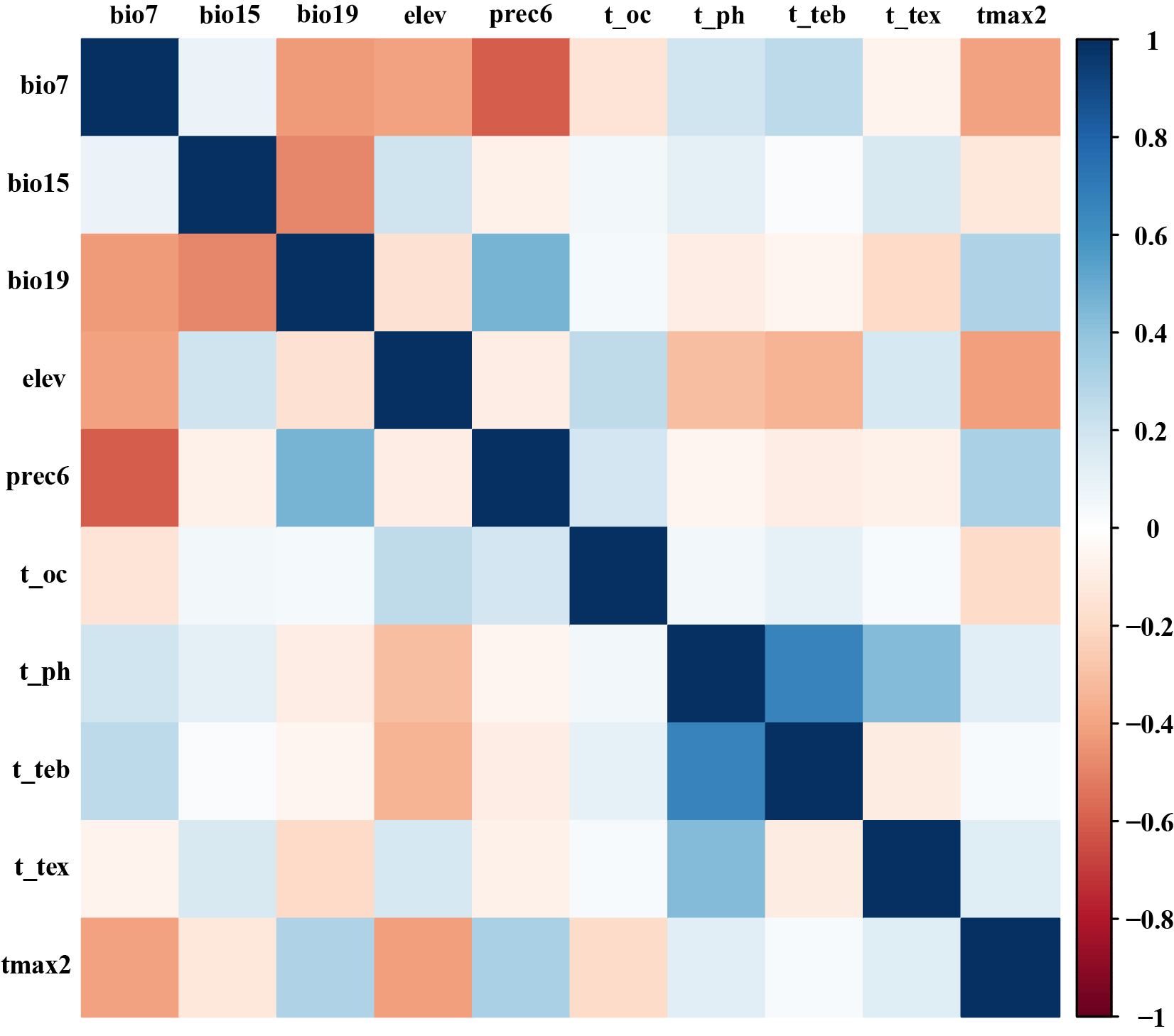

Supplement: Supplementary file 1 [file Image_1.JPEG]

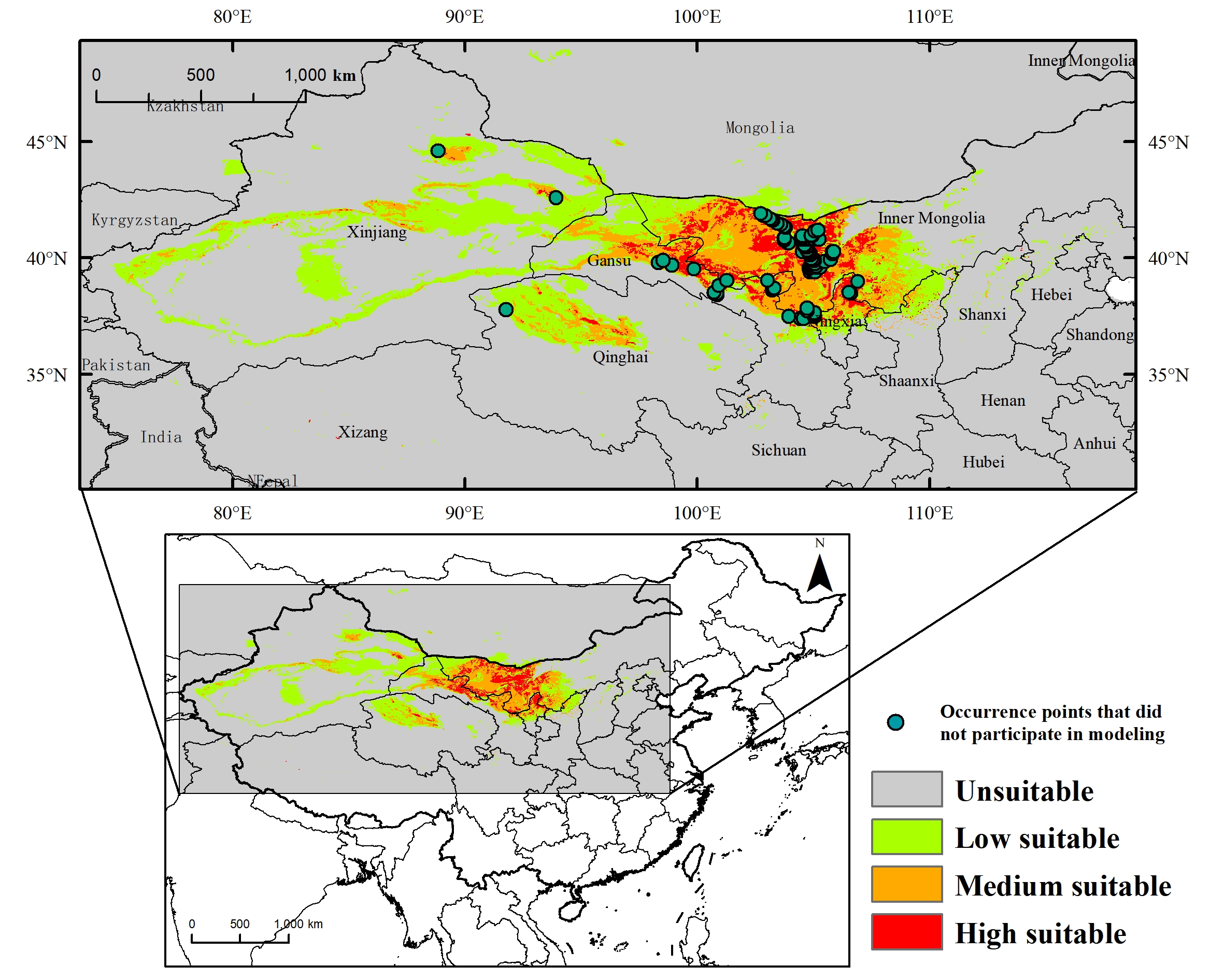

Supplement: Supplementary file 2 [file Image_2.JPEG]

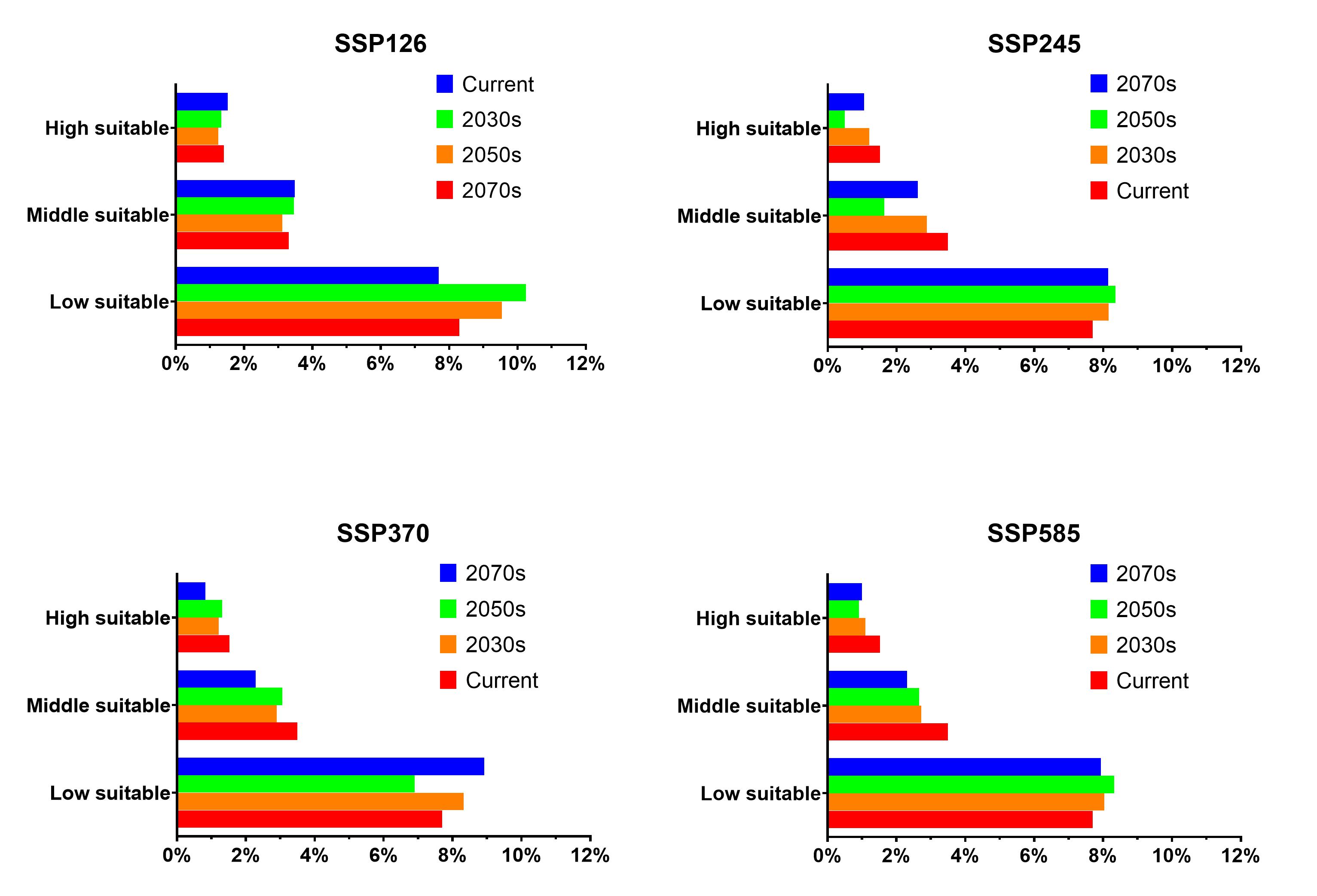

Supplement: Supplementary file 3 [file Image_3.JPEG]
