# Supplementary material for: Predicting the distribution of suitable habitat of the poisonous weed Astragalus variabilis in China under current and future climate conditions
Source: Front Plant Sci. 2022 Sep 9;13:921310. doi: 10.3389/fpls.2022.921310 (PMC9531759; doi:10.3389/fpls.2022.921310)
Supplement: Supplementary file 5 [file Table_2.DOCX]

**Table S2 Environmental variables and their type and description.**

| **Environmental variable type (source)** | **Variable** | **Description** | **Unit** |
| --- | --- | --- | --- |
| **Bioclimatic**  **(Worldclim v2.1)** | **bio1** | **Annual mean temperature** | **℃** |
|  | **bio2** | **Mean diurnal range** | **℃** |
|  | **bio3** | **Isothermality** | **/** |
|  | **bio4** | **Temperature seasonality** | **Cof V** |
|  | **bio5** | **Max temperature of warmest month** | **℃** |
|  | **bio6** | **Min temperature of coldest month** | **℃** |
|  | **bio7** | **Temperature annual range** | **℃** |
|  | **bio8** | **Mean temperature of wettest quarter** | **℃** |
|  | **bio9** | **Mean temperature of driest quarter** | **℃** |
|  | **bio10** | **Mean temperature of warmest quarter** | **℃** |
|  | **bio11** | **Mean temperature of coldest quarter** | **℃** |
|  | **bio12** | **Annual precipitation** | **mm** |
|  | **bio13** | **Precipitation of wettest month** | **mm** |
|  | **bio14** | **Precipitation of driest month** | **mm** |
|  | **bio15** | **Precipitation seasonality** | **/** |
|  | **bio16** | **Precipitation of wettest quarter** | **mm** |
|  | **bio17** | **Precipitation of driest quarter** | **mm** |
|  | **bio18** | **Precipitation of warmest quarter** | **mm** |
|  | **bio19** | **Precipitation of coldest quarter** | **mm** |
| **Climate**  **(Worldclim v2.1)** | **tmin1~12** | **Monthly average minimum temperature** | **℃** |
|  | **tmax1~12** | **Monthly average maximum temperature** | **℃** |
|  | **prec1~12** | **Monthly total precipitation** | **mm** |
| **Topography**  **(Worldclim v2.1)** | **elev** | **Elevation** | **m** |
| **Soil**  **（HWSD v1.2）** | **t_oc** | **Topsoil organic carbon** | **% weight** |
|  | **t_ph** | **Topsoil pH (H_2_O)** | **-log(H^+^)** |
|  | **t_teb** | **Topsoil total exchangeable bases** | **cmol/kg** |
|  | **t_tex** | **Topsoil USDA texture** | **/** |
