# Supplementary material for: Predicting the distribution of suitable habitat of the poisonous weed Astragalus variabilis in China under current and future climate conditions
Source: Front Plant Sci. 2022 Sep 9;13:921310. doi: 10.3389/fpls.2022.921310 (PMC9531759; doi:10.3389/fpls.2022.921310)
Supplement: Supplementary file 6 [file Table_3.DOCX]

**Table S3 Livestock poisoning occurs of** ***A. variabilis***

| **Year** | **Description of Livestock poisoning occurs place （Chinese and English）** | **Reference /information source** |
| --- | --- | --- |
| 1970-1974 | 内蒙古阿拉善左旗巴音诺尔公  Bayinuoergong, Alxa Left Banner, Inner Mongolia | [1] |
| 1972 | 内蒙古阿拉善左旗温都尔勒图  Wenduerletu, Alxa Left Banner, Inner Mongolia | [1] |
| 1976 | 内蒙古阿拉善左旗乌力吉  Wuligi, Alxa Left Banner, Inner Mongolia | [2] |
| 1976-1978 | 甘肃张掖市民乐县  Minle County, Zhangye City, Gansu Province | [3] |
| 1979 | 内蒙古阿拉善左旗庆格勒图  Qinggeletu, Alxa Left Banner, Inner Mongolia | [2] |
| 1978-1979 | 内蒙古鄂尔多斯鄂托克旗三北羊场  Sanbei Sheep Farm of Etoke Banner, Ordos City, Inner Mongolia Autonomous Region | [2] |
| 1979 | 内蒙古阿拉善左旗罕乌拉  Hanwula, Alxa Left Banner, Inner Mongolia | [4] |
| 1984 | 内蒙古阿拉善右旗萨日台  Saretai Sumu, Alxa Right Banner, Inner Mongolia | [4] |
| 1987-1988 | 甘肃国营黄羊河牧场  Gansu state - owned Huangyang River pasture | [5] |
| 1988 | 宁夏陶乐镇  Former Taole County of Ningxia Hui Autonomous Region | [3] |
| 1982-1992 | 内蒙阿拉善  Alxa, Inner Mongolia | [4] |
| 1992-1995 | 甘肃马鬃山地区  Manong Mountain area, Jiuquan City, Gansu Province | [6] |
| 2003 | 内蒙古阿拉善左旗巴彦洪格日  Bayanhonggeri, Alxa Left Banner, Inner Mongolia | [7] |
| 2003-2005 | 内蒙古阿拉善左旗  Alxa Left Banner, Inner Mongolia | [2] |
| 2004 | 甘肃民勤县  Minqin county of Gansu Province | [8] |
| 2010 | 内蒙古阿拉善左旗乌力吉  Wulji Sumu, Alxa Left Banner, Inner Mongolia | [9] |
| 2005-2012 | 内蒙古阿拉善左旗吉兰泰  Jilantai, Alxa Left Banner, Inner Mongolia | [10] |
| 2015-2017 | 新疆巴里坤县盆地草原  Grassland of Santanghu Basin, Balikun County, Xinjiang | [11] |
| 2006 | 内蒙古阿拉善左旗[温都尔勒图镇](https://baike.baidu.com/item/%E6%B8%A9%E9%83%BD%E5%B0%94%E5%8B%92%E5%9B%BE%E9%95%87)  Wenduerletu, Alxa Left Banner, Inner Mongolia | Field survey |
| 2010 | 内蒙古阿拉善左旗额尔克哈什哈  Erekehashenha, Alxa Left Banner, Inner Mongolia | Field survey |
| 2018 | 新疆哈密往马鬃山方向  Xinjiang Hami to Ma Manong Mountain area | Field survey |
| 2000-至今 | 内蒙古阿拉善左旗银根  Yingen, Alxa Left Banner, Inner Mongolia | Field survey; [12]; News reports （http://news.sohu.com/20041118/n223062761.shtml)(https://news.sina.com.cn/o/2005-08-09/11326648039s.shtml） |
| 2021 | 甘肃金塔县以北  North of Jinta County, Gansu Province | Field survey |
| 2022 | 内蒙古阿拉善左旗嘉尔格勒赛汉  Jiaergelesaihan, Alxa Left Banner, Inner Mongolia | Field survey |

Note:Specific coordinates are used if there are specific coordinates in the literature. If the literature only describes the location, we use the Baidu coordinate pickup system to search. Logistic values are extracted by region at county level and above, and by coordinate below county level.

**References**

[1] Alxa Left Banner Agricultur Husbandry and Forestry Science and Technology Service Station. Experimental results of eradicating poisonous grass in grassland by chemical agents [J]. Ningxia Agricultural Science and Technology, 1974, (1): 25-33.

阿拉善左旗农牧林科技服务站. 利用化学药剂铲除草原毒草试验结果 [J]. 宁夏农业科技, 1974: 25-33. [In Chinese]

[2] Da N T. Research history of Locoweed in Alxa Left Banner [J]. Animal husbandry and Veterinary Abstracts of China, 2016, 32(6): 43-4.

达能太. 阿拉善左旗疯草研究历程 [J]. 中国畜牧兽医文摘, 2016, 32 (6): 43-4. [In Chinese]

[3] Zhao B Y. The systematic analysis and toxic comparative pathology research of locoweed (Oxytropis kansuensis) alkaloid [D]; Northwest Agriculture & Forestry University, 2001.

赵宝玉. 疯草（甘肃棘豆）生物碱系统分析及其毒性的比较病理学研究 [D]. 陕西杨凌; 西北农林科技大学, 2001. [In Chinese]

[4] Chen S K, Sa R. Investigation on the distribution and harm status of poisonous grass in desert grassland of Alxa League [J]. Grassland of China, 1992, (3): 60-2.

陈善科, 萨仁. 阿拉善盟荒漠草场毒草分布及其危害现状的调查 [J]. 中国草地, 1992, 1992 (3): 60-2. [In Chinese]

[5] Zhao B Y. Toxic plants of Astragalus in China and their harm to domestic animals [J]. Chinese Journal of Veterinary Medicine, 1994, 20(4): 15-6.

赵宝玉. 我国黄芪属有毒植物及其对家畜的危害 [J]. 中国兽医杂志, 1994, (4): 15-6. [In Chinese]

[6] Muhanmode A, Cheng H W. Incestigation on Poisonous Astragalus Variabilis and Its Damaging Status in Natural Grassland Throughout Mazong Mountain [J]. Pratacultural Science, 1999, 16(1): 49-51+4.

穆罕默德·阿不来提, 程宏伟. 马鬃山一带天然草地毒草变异黄芪及其危害现状的调查研究 [J]. 草业科学, 1999, (1): 49-51. [In Chinese]

[7] Zhang Y H, Pan L F, Yang W K, et al. Investigation and prevention and cure of Astragalus variabilis poisoning in Bayan Honggeri Sumu sheep [J]. Progress in Veterinary Medicine, 2005, (4): 118.

张有辉, 潘发利, 杨万科, 等. 巴彦洪格日苏木羊变异黄芪中毒病的调查及防治 [J]. 动物医学进展, 2005: 118. [In Chinese]

[8] Tan C J, Dong Q, Zhao B Y, et al. The investigation of poisonous weeds on natural grassland in Minqin County[J]. Pratacultural Science,2005(03):86-89.

谭承建,董强,赵宝玉,等.民勤县天然草地毒草调查与防治[J].草业科学,2005(03):86-89. [In Chinese]

[9] Wei J H, Xu X J, Sha R F. Review of the prevention and control of locoweed poisoning in livestock [J]. Chinese livestock and poultry seed industry, 2011, 7(8): 36-7.

魏菊红, 徐向军, 沙日夫. 家畜“疯草”中毒病防治工作的回顾 [J]. 中国畜禽种业, 2011, 7(8): 36-7. [In Chinese]

[10] Zhang S Y, Aoyun G R L, Wang D D. Investigation and prevention of locoweed poisoning in livestock in Jilantai region [J]. Animal husbandry and Veterinary Abstracts of China, 2012,28(01):81.

张淑英,敖云格日勒,王丹丹.吉兰太地区家畜疯草中毒的调查及防治[J].中国畜牧兽医文摘,2012,28(01):81. [In Chinese]

[11] Zhao G W, Gu L J. Investigation on Wild Poisonous Plant Resources in Barikun County Xinjiang[J]. Grass-feeding Livestock,2018(03):54-59.

赵国伟,谷林俊.新疆巴里坤县野生有毒植物资源调查研究[J].草食家畜,2018(03):54-59. [In Chinese]

[12] Li G Z, Morigen B L G, Sha R K, et al. Study on occurrence regularity and prevention and control technology of main toxic grass in Alxa grassland[J]. Contemporary livestock and poultry farming,2019(10):13-14+7.

李国中,莫日根別力格,沙日扣,等.阿拉善草原主要毒害草发生规律及防控技术研究[J].当代畜禽养殖业,2019(10):13-14+7. [In Chinese]
